# Homologous prime-boost immunization with live attenuated *Salmonella enterica* serovar Senftenberg and its preventive efficacy against experimental challenge with various strains of *S.* Senftenberg

**DOI:** 10.1186/s12917-017-0960-4

**Published:** 2017-01-31

**Authors:** Nitin M. Kamble, John Hwa Lee

**Affiliations:** College of Veterinary Medicine and Bio-Safety Research Institute, Chonbuk National University, Iksan Campus, 570-752 Republic of Korea

**Keywords:** *Salmonella* Senftenberg, Mucosal immunity, Persistence, Fecal shedding

## Abstract

**Background:**

The heterogeneity observed regarding persistence, and subsequent fecal shedding pattern of the *Salmonella* Senftenberg (*S.* Senftenberg) serovar in chicken’s calls for development of the optimized immunization strategy which can provide protection against various *S.* Senftenberg isolated. Optimization of an immunization strategy with a live attenuated *S.* Senftenberg *(Δlon and ΔcpxR)* vaccine candidate (JOL1587) was undertaken in this study to evaluate the ability of a homologous prime-boost immunization strategy (using JOL1587) to confer protection against four different *S.* Senftenberg isolates in chickens.

**Results:**

After oral immunization with JOL1587, the humoral, mucosal and cell-mediated immune responses were significantly higher in double immunized chickens than in single immunized and control group chickens. A significant increase in the multifunctional cytokine IL-6 and in helper and cytotoxic T cell populations after a booster immunization also indicated the advantage of double over single immunization. The four different *S.* Senftenberg field isolates were characterized by their persistence levels in chickens, and were subsequently used for challenge experiments to evaluate the differences in protective efficacy conferred by single and double immunization. Chickens from the doubleimmunized group exhibited significant reduction in the shedding of all four wild-type *S.* Senftenberg challenge strains below the detection limit in the fecal samples. Single immunized chickens showed a decrease in fecal shedding, but failed to exhibit complete protection against all the challenge strains.

**Conclusion:**

Although single immunization with JOL1587 showed a reduction in the fecal shedding of challenge strains, only the homologous prime-boost immunization strategy provided an adequate immune response for increased protection against all four challenge strains of *S.* Senftenberg from the feces of chickens.

## Background

The nontyphoidal serovars of the gram-negative pathogen *Salmonella* enterica are a leading cause of invasive bacterial gastroenteritis in humans [1]. According to the Centers for Disease Control and Prevention (CDC), there are 94 million cases of nontyphoidal gastroenteritis and 115,000 deaths reported annually in the worldwide human population. The *Salmonella* enterica subsp. Enterica serovar Senftenberg (*S.* Senftenberg) is one of the nontyphoidal serotypes (NTS) that was isolated from several outbreaks of gastroenteritis in humans [2–4]. *S.* Senftenberg is a gram-negative, facultative, intracellular pathogen affecting both poultry and humans [5, 6]). The *Salmonella* pathogen is transmitted to humans either through direct contact with an infected animal or by consumption of contaminated food and water. The serovar *S.* Senftenberg is primarily associated with hatcheries, but recent reports have suggested its emergence and persistence in poultry environments throughout the rearing season. This could pose a potential risk of contamination of poultry products [7, 8]. Moreover, *S.* Senftenberg cells are reported to tolerate desiccation and survive routine cleaning and disinfection procedures, enabling them to persist longer and transmit more easily to other poultry and humans [8]. The hallmark of *S.* Senftenberg infection in chickens is its high colonization of the cecum, which in turn leads to an asymptomatic carrier state in chickens [7]. Various strains of *S.* Senftenberg isolated from different poultry outbreaks were analyzed for their persistence and fecal shedding patterns in the cecum [9]. As previously reported, a coordinated immune response from the systemic and the mucosal arms of the immune system is needed for clearance of *Salmonella* infection [10]*.* Therefore, generation of both a systemic and a mucosal immune response locally in the intestine is essential for the control of *S.* Senftenberg colonization and fecal shedding.

Homologous immunization with a *S.* Senftenberg vaccine can be an approach for managing persistent infection of *S.* Senftenberg in hatcheries as well as poultry farms. Recently, we developed a live attenuated *(Δlon and ΔcpxR) S.* Senftenberg vaccine candidate strain (JOL1587) to provide protection against *S.* Senftenberg [11]. The fact that different *S.* Senftenberg strains exhibit variation in the fecal shedding pattern and extent of colonization in the cecum underscores the need for optimization of the immunization strategy with JOL1587. The principle objective of this study was to generate a robust systemic and mucosal immune response to prevent fecal shedding of the persistent and the non-persistent *S.* Senftenberg strains. We evaluated the mucosal and systemic immune response generated in chickens following single or double immunization with a live attenuated mutant of *S.* Senftenberg (*∆lon* and *∆cpxR*). The ability to confer a protective immune response by mucosal immunization was evaluated by assessing the rate of fecal shedding and persistence of four different wild-type strains of *S.* Senftenberg in challenged chickens.

## Methods

### Preparation of the mutant and challenge strain

The attenuated mutant of *S*. Senftenberg JOL1587 was constructed by deleting the *lon* and *cpxR* genes from wild-type S. Senftenberg JOL1556 by the allelic exchange method described previously [11, 12]. Four strains of *S*. Senftenberg (JOL1557, JOL1815, JOL1816 and JOL1817) isolated from different field outbreaks in chickens and characterized for persistence in the caecum were used for the challenge study. The wild-type strains were characterized for presence of the specific pathogenicity island (SPI) related genes by PCR (Table 1). All bacterial strains were cryopreserved as frozen glycerol cultures in Luria-Bertani (LB) broth at−80 °C. The glycerol stocks were revived by streaking the thawed cultures on LB agar and incubating at 37 °C for 24 h. A single colony was picked from overnight grown culture, inoculated in LB broth at 1:20 dilution, and grown in shaker culture until the optical density at 600 nm (OD_600_) reached 0.6. The culture was adjusted to a suitable concentration before inoculation into chickens.

**Table 1:** Fecal shedding of wild-type *S*. Senftenberg from challenged chickens

Challenge Strain	Group	Subgroup	Days post inoculation						
			1	3	7	9	14	21	28
JOL1557	A	A1	100^a^	100	100	100	100	100	100
	B	B1	100	60	40	40	40	40	40
	C	C1	100	60	40	40	0	0	0
JOL1815	A	A2	100	100	100	100	100	80	80
	B	B2	100	40	60	40	60	60	40
	C	C2	100	60	40	10	0	0	0
JOL1816	A	A3	100	100	100	100	100	100	100
	B	B3	100	40	0	40	40	60	60
	C	C3	100	60	40	40	0	10	0
JOL1817	A	A4	100	100	100	100	100	80	80
	B	B4	100	40	60	40	60	40	0
	C	C4	100	60	40	10	0	0	0

A: PBS inoculated group; B: Prime immunized group and C: Prime-boost immunized group

Each major group A, B and C are further sub-divided into four subgroups and challenged with JOL1557, JOl1815, JOL1816 and JOL1817, respectively

^a^Percentage population tested positive for recovery of the *S.* Senftenberg in fecal samples

### Chicken immunization and challenge experiments

For chicken experimentation, female day-old layer chickens (Brown Nick, *n* = 75) were procured from JOINBIO hatchery located in the yongin-si, South Korea. The chickens were divided into three groups (*n* = 25). The strategy for the immunization and challenge study is explained in Table 2. All animals were provided with ad libitum antibiotic-free feed and water. At the fourth week of age, group A was inoculated orally with PBS to serve as a non-immunized control and groups B and C were inoculated orally with a JOL1587 suspension containing 1 × 10^8^ colony forming units (CFUs). Further, group C was given a booster dose orally at the 8 week of age with a JOL1587 suspension containing 1 × 10^8^ CFUs. Post-immunization and booster, the serum and intestinal lavage samples were collected from six randomly selected chickens from the pool of 25 chickens in each group.

**Table 2:** Immunization and challenge strategy

Group	N	Immunization		Subgroups	Animal No.	Challenge strain
		Primary	Booster			
A	25	PBS	PBS	AI	6	JOL1557
				AII	6	JOL1815
				AIII	6	JOL1816
				AIV	6	JOL1817
B	25	JOL1587	PBS	BI	6	JOL1557
				BII	6	JOL1815
				BIII	6	JOL1816
				BIV	6	JOL1817
C	25	JOL1587	JOL1585	CI	6	JOL1557
				CII	6	JOL1815
				CIII	6	JOL1816
				CIV	6	JOL1817

### Collection of the intestinal wash for sIgA

The intestinal lavage samples were collected for measurement of the secretory IgA levels according to a protocol described elsewhere [13]. Briefly, chickens were taken off feed for 16 h. The off-feed chickens were orally administered 5 mL of lavage solution (0.2 M Na_2_SO_4_, 0.2 M NaHCO_3_, 0.1 M KCl, 0.25 M NaCl, and 16.25% polyethylene glycol in distilled water) and kept in clean, disinfected buckets. Thirty minutes post-administration, the chickens were injected intramuscularly with 200 μl of a 5% pilocarpine solution. The chickens were observed for mucinous droppings over a period of thirty minutes. The mucinous droppings were collected in microcentrifuge tubes containing 1× soybean trypsin protease inhibitor and 50 mM EDTA solution. Samples were preserved by adding 10 μL each of 5% bovine serum albumin, 10% sodium azide and 1% PMSF (phenylmethylsulfonyl fluoride). Samples were stored at−20 °C until analysis.

### Enzyme-linked immunosorbent assay (ELISA)

The development of *S.* Senftenberg-specific humoral and mucosal immunity in response to JOL1587 immunization in chickens was evaluated by estimating the variation in plasma IgG and intestinal sIgA levels by *S.* Senftenberg antigen-specific indirect ELISA. The outer membrane protein fraction (OMP) was extracted from wild-type *S.* Senftenberg as described elsewhere (Osborn and Munson, 1974). The levels of OMP specific IgG antibodies in the plasma of the immunized chickens were determined using an indirect ELISA. Briefly, 96-well Microlon® ELISA plates (Greiner Bio-One GmbH, Fricken hausen, Germany) were coated with OMP (50 μg/mL) and blocked with 5% skim milk powder in PBS. For total IgG and sIgA detection, the ELISA plates were incubated with an intestinal wash (1:5 dilution) and plasma (1:100 dilution) and then incubated with horseradish peroxidase (HRP)-conjugated goat anti-chicken IgG and IgA at a 1:100,000 dilution for 1 h. The plates were developed with o-phenylenediamine dihydrochloride substrate (Sigma-Aldrich, St. Louis, MO, USA) and read at 492 nm.

### Lymphocyte proliferation assay (LPA)

The LPA stimulation indices were calculated 3 weeks post-prime- and –booster immunization to estimate the cellular immune response generated after JOL1587 immunization in chickens. From each group (A, B and C) heparinized blood samples were collected from six randomly selected chickens from the pool of 25 chickens. Cells were stimulated with soluble antigen prepared from the *S.* Senftenberg wild-type strain, as described elsewhere [14]. Soluble antigen was prepared from the wild-type *S.* Senftenberg strain JOL1556. Briefly, the overnight grown bacterial cell culture were pelleted and resuspended in PBS. The bacterial suspension was sonicated for 5 min, and pelleted by centrifugation at 5000 × *g* for 60 min at 4 °C. The supernatant containing the sonicated bacterial cell protein suspension (sbcp) was collected and used as the soluble antigen. Post-inoculation, the peripheral blood mononuclear cells (PBMCs) were separated from the heparinized blood collected from the jugular veins of five randomly selected chickens per group. Blood was carefully loaded on Histopaque-1083 (Sigma-Aldrich, St. Louis, MO, USA) and centrifuged at 400 × g for 40 min at room temperature. The buffy coat at the interface was suspended in RPMI-1640 medium (HyClone Laboratories, South Logan, UT, USA). The PBMCs were adjusted to a 1 × 10^6^ cells/mL final concentration in the RPMI-1640 medium and distributed in triplicate to 96-well tissue culture plates. The plates were incubated with medium alone or medium containing 4 μg/mL of the soluble antigen of *S.* Senftenberg at 40 °C with 5% CO_2_ for 72 h. Cell proliferation was measured by using thiazolyl blue tetrazolium bromide (MTT) dye (Sigma-Aldrich, St. Louis, MO, USA) as per the manufacturer’s protocol. The blastogenic response was expressed as mean stimulation index (SI), which was calculated as the ratio of the average OD of the stimulated/unstimulated wells.

### Flow cytometry

Post-booster immunization, variations in the JOL1587-specific helper (CD3 + CD4+) and cytotoxic (CD3 + CD8+) T cell populations were analyzed following ex vivo stimulation of peripheral blood mononuclear cells (PBMCs) with soluble antigen extracted from wild-type *S.* Senftenberg. PBMCs were separated from blood samples of five chickens from each group (A, B and C) at 1 week post-prime and booster immunization. The isolated PBMCs were stimulated ex vivo with soluble antigen extracted from wild-type *S.* Senftenberg. The stimulated PBMCs were stained with anti-CD3, anti-CD4 and anti-CD8 antibodies, as described elsewhere [15]. Briefly, a total of 1 × 10^6^ PBMCs were stained with appropriately diluted mouse anti-chicken CD3-FITC, CD4-APC and CD8-RPE (Southern Biotech, USA). After incubation, all stained samples were washed three times with PBS and analyzed with a flow cytometer (Miltenyi Biotech, Germany). A total of 10,000 events were recorded and analyzed using the FlowJo single cell analysis software.

### Relative quantification of cytokines by real-time quantitative reverse transcriptase PCR (qRT-PCR)

The cytokine response was evaluated after prime and booster immunization in PBMCs following ex vivo stimulation with the soluble antigen of *S.* Senftenberg. The qRT-PCR method was employed for relative quantification of IL-6, IFN-γ, and IL-2 cytokine mRNA levels. The RNA was isolated from PBMCs separated at ten days post prime and booster vaccination. The housekeeping gene β-actin was used as an endogenous control for normalization of the sample Ct values. The comparative 2^-∆∆Ct^ method was used for the relative quantification of the fold change in the cytokine mRNA levels [16].

### Fecal shedding of the different strains of S. Senftenberg

At the twelve week of age, chickens from each group were further separated into four sub-groups containing six chickens each, and orally challenged individually with virulent wild-type (WT) *S.* Senftenberg strain, JOL1557, JOL1815, JOL1816, and JOL1817 (5 × 10^8^ CFUs), respectively. For fecal shedding, the fecal samples were collected from six chickens in each sub-group using sterile, buffered peptone water (BPW, Becton Dickinson, USA). A ten-fold dilution of BPW was plated onto brilliant green agar (BGA, Becton Dickinson) and incubated at 37 °C for 24 h. Samples showing colonies on BGA were counted as positive. Negative samples were further incubated in Rappaport-Vassiliadis R10 enrichment broth (RV, Becton Dickinson) for 48 h at 42 °C. The enriched culture was picked with a sterile loop and then plated onto BGA. Samples that were positive after direct plating or after enrichment were taken as positive for harboring *S.* Senftenberg. The challenge strain was confirmed by PCR using *S.* Senftenberg serotype-specific primers. The challenge strain was distinguished from inoculated JOL1587 (*∆lon/∆cpxR)* by using primer sets specific to *lon* and *cpxR* genes. The enumeration of the cfu per gram of tissue was performed as described elsewhere [17].

### Statistical analysis

The results of this study are expressed as means ± SEM unless otherwise specified. A one-way ANOVA with Bonferroni corrections was employed to analyze statistical significance for expression levels of CD3 + CD4+ T cells, CD3 + CD8+ T cells, cytokines, plasma IgG and intestinal sIgA. The values for the individual groups were considered statistically significant if the *P*-values were ≤0.05 or ≤ 0.01. The fecal shedding data was converted into percentage value for comparison. All analysis was performed using the SPSS 16.0 (SPSS Inc., USA).

## Results

### Characterization of wild type S. Senftenberg strain

Four wild-type *S*. Senftenberg strains, JOL1557 (WT), JOL1815 (WT), JOL1816 (WT) and JOL1817 (WT) were characterized by PCR for presence of various SPI specific genes (Table 3). The PCR data indicated the size-specific amplification and presence of the SPI1, 2, 3, 4 and 5 specific analyzed genes in all four wild-type *S.* Senftenberg strain (Data not shown). The four groups of the non-immunized chickens were inoculated separately with JOL1557 (WT), JOL1815 (WT), JOL1816 (WT) and JOL1817 (WT) for evaluation of the persistence in the cecum. The bacterial recovery experiment showed that all the four wild-type strains were recovered from cecum swabs on BGA plates until week four post-inoculation.

**Table 3:** List of primers used for detection of genes specificto different SPI

SPI	Primers	Sequence 5'–3'	Amplicon Size (bp)
SPI1	invAE F	CAGCGATATCCAAATGTTGC	2168
	invAE R	AAATGGCAGAACAGCGTCGTA	
	hilA F	CTGCCGCAGTGTTAAGGATA	497
	hilA R	CTGTCGCCTTAATCGCATGT	
	avr F	AGACTTATATTCAGCTATCC	1115
	avr R	ACATAACCCTGCTGTACCTG	
SPI2	aa permease F	ACCATTCAAGAGACAATTGG	1737
	aa permease R	GTCCTGTTCTGGTATTACGC	
SPI3	mgtC F	ATGAATCCCCAAAATTAAGG	1153
	mgtC R	AATCATCTGGCAAGTTAACG	
SPI4	ABC Trans F	CAGTCTATCACAGCAAGGCA	1409
	ABC Trans R	TTATCCGGAGAACAATCACG	
SPI5	pipB F	AATATTGGATGGGGGAAAAG	230
	pipB R	AACCTGACTCACGCAGACCT	

### Estimation of S. Senftenberg-specific humoral and mucosal immune responses

**Fig. 1:**
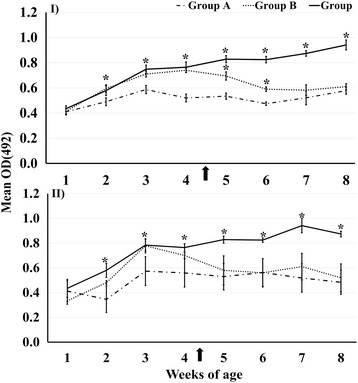
Graphical representation of the OD values for evaluation of the plasma IgG and intestinal lavage sIgA responses to JOL1587 immunization in chickens. **I**) Plasma IgG levels, **II**) Intestinal lavage sIgA levels. a: PBS-inoculated group; b: single immunized group and c: double immunized group. The arrow denotes the time of booster immunization in group C chickens. Values shown for each group are the mean ± SEM of antibody levels (*n* = 6). The * symbol indicates a significant increase in the antibody levels in the JOL1587 immunized chickens compared to the control group chickens (*P* ≤ 0.05)

Indirect ELISA estimated differences in humoral and mucosal immune responses generated after single and double immunization. Immunization of the chickens with JOL1587 led to a significant rise (*P* ≤ 0.05) in the IgG and sIgA levels in both the single and double immunized groups relative to the control group chickens (Fig. 1). The effect of booster immunization was apparent for both the plasma IgG and intestinal sIgA responses in that levels of each increased significantly (*P* ≤ 0.05) only in the double immunized group. The chickens from the single immunized group (coinciding with the timing for booster immunization) started exhibiting sharp and significant declines (*P* ≤ 0.05) in both IgG and sIgA levels compared to the double immunized group. The levels of IgG and sIgA in the single immunized group became insignificant and similar to control group chickens by eleven weeks of age (*P* ≤ 0.05, Fig. 1). The non-immunized control group chickens showed insignificant rise in *S.* Senftenberg-specific IgG and sIgA levels throughout the observation period (Fig. 1).

### Evaluation of S. Senftenberg-specific cellular immune responses

**Fig. 2:**
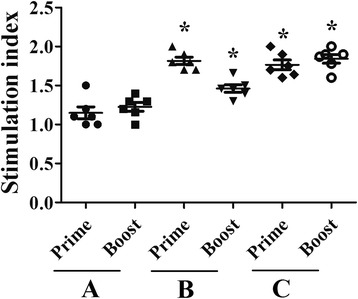
Stimulation indices for measurement of a lymphocyte proliferation response in the JOL1587-immunized and control group chickens. **a** PBS-inoculated group; **b** Single immunized group and **c**: Double immunized group. The LPA assay was performed at 7 and 11 weeks of age after the prime and booster immunizations, respectively. The * symbol indicates a significant increase in the lymphocyte proliferation response in the JOL1587 immunized chickens compared to the control group chickens (*P* ≤ 0.05). Total six samples were analyzed which were collected from six randomly selected chickens from group a, b and c

As shown by the LPA results, the immunization of chickens with JOL1587 successfully induced an *S.* Senftenberg-specific cellular immune response in both single and double immunized groups (Fig. 2). For calculation of significant differences in the cellular immune response, the SI value for an immunized group was compared with control group chickens. The LPA results showed that the cellular immune response in the double immunized group was significantly elevated (*P* ≤ 0.05) after prime (1.6-fold) as well as booster 2.0-fold) immunization (Fig. 2). The single immunized chickens, which showed a significant rise (1.7-fold, *P* ≤ 0.05) in SI value after prime immunization, contrastingly showed an insignificant decline in the SI value (1.5-fold, *P* ≤ 0.05) at eleven week of age.

### Analyses of helper and cytotoxic T cell populations

**Fig. 3:**
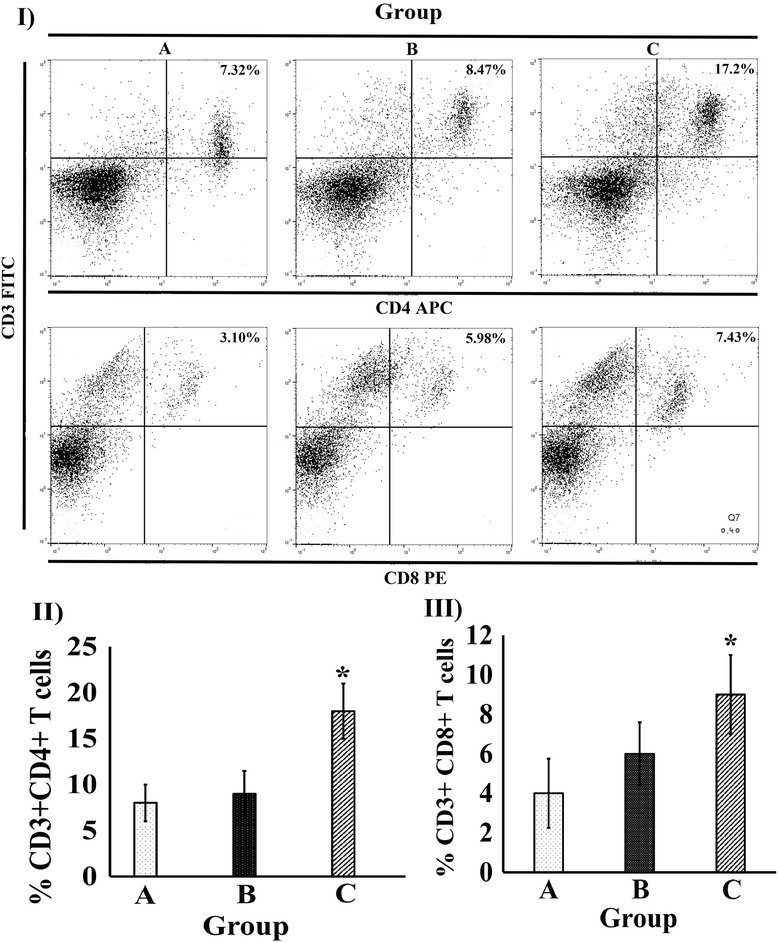
FACS analysis for peripheral CD3 + CD4+ and CD3 + CD8+ T cell populations in the JOL1587-immunized and control group chickens. The isolated PBMCs were stained with appropriately diluted fluorescein-isothiocyanate (FITC)-labeled anti-CD3, allophycocyanin (APC)-labeled anti-CD4, and phycoerythrin (PE)-labeled anti-CD8 monoclonal antibodies. *a*: PBS-inoculated group; *b*: single immunized group and *c*: double immunized group. (**I**) Representative dot plots for CD3 + CD4+ and CD3 + CD8+ T cell populations. The population is represented as a percentage of gated cells; (**II**) The percentage of peripheral CD3 + CD4+ T cells; (**III**) The percentage of peripheral CD3 + CD8+ T cells. The values are shown as the mean ± SEM of five chickens per group. The * symbol indicates a significant increase in the percentage of peripheral T lymphocytes in the JOL1587-immunized chickens compared to the control group chickens (*P* ≤ 0.05)

For estimation of the differences in the helper and cytotoxic T cell populations, the CD3 + CD4+ and CD3 + CD8+ T cell populations for the immunized groups were compared with control group chickens. Both the single and double immunized chickens showed a significant increase (*P* ≤ 0.05) in cytotoxic T cell populations after prime-immunization. However, this failed to generate a significant increase in the peripherally circulating helper T cell populations as compared to the control group chickens (Fig. 3I). Our experimental data showed that the significant increase (*P* ≤ 0.05) in both the helper and cytotoxic T cell populations was observed in the double immunized group only after the booster immunization (Fig. 3I). The quantitative estimation of the CD3+ CD4+ and CD3+ CD8+ T cell populations in the double immunized chickens showed these were approximately 2.5- and 1.5- fold higher than in the control group chickens, respectively (*P* ≤ 0.05, Fig. 3II). After booster immunization, the levels of the CD3 + CD4+ and CD3+ CD8+ T cell populations for the single immunized and control group chickens were similar, with no fold change differences observed (Fig. 3).

### Cytokine mRNA expression levels

**Fig. 4:**
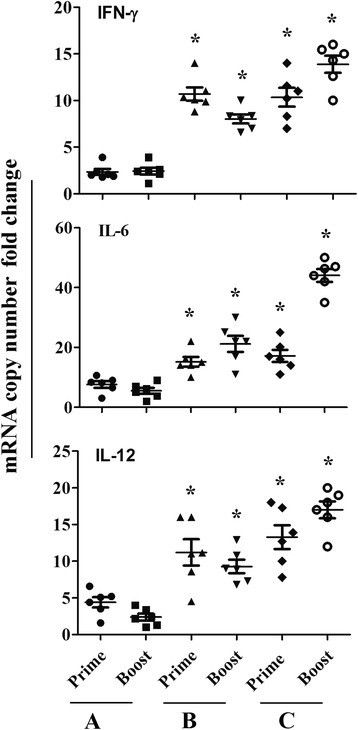
The IFN-γ, IL-2 and IL-6 cytokine profiles of in vitro*-*stimulated PBMCs from the JOL1587-immunized and control group chickens. The relative mRNA copy numbers were determined by qRT-PCR using the comparative Ct method. I) Prime inoculation with JOL1587 at the fourth week of age and II) Prime-boost inoculation with JOL1587 at the 8 week of age. **a** PBS-inoculated group; **b** Prime immunized group and **c** Prime-boost immunized group. The * symbol indicates a significant increase in the cytokine levels for the JOL1587-immunized group compared to the control group chickens (*P* ≤ 0.05).Total six splenocytes samples were analyzed which were collected from six randomly selected chickens from group a, b and c

The fold changes observed in the mRNA copy number of the IL-6, IFN-γ and IL-2 cytokines in the PBMCs from control and immunized group chickens are shown in Fig. 4. Both the single and double immunized groups showed significant upregulation (*P* ≤ 0.05, Fig. 4) of the IL-6, IFN-γ and IL-2 cytokines relative to control group chickens. After quantitative estimation, IFN-γ and IL-2 cytokine mRNA expression levels were upregulated 10- and 16-fold in the single and double immunized groups, respectively (*P* ≤ 0.05, Fig. 4). IL-6 showed a 16- and 24-fold increase in the single immunized group and a 16- and 46-fold increase in the double immunized group (*P* ≤ 0.05, Fig. 4).

### Protective efficacy against different wild-type strains of S. Senftenberg

**Fig. 5:**
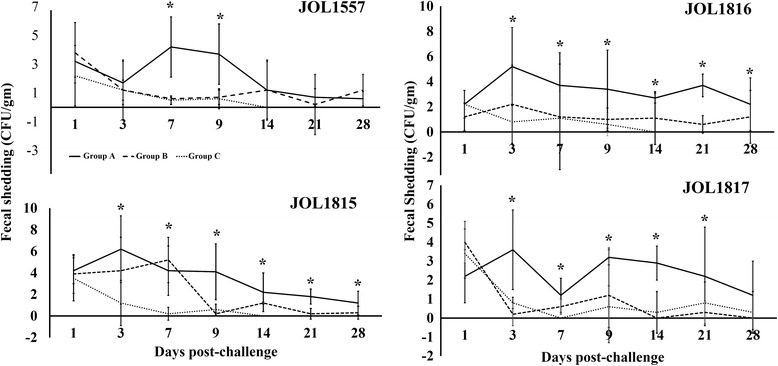
Faecal shedding level of different *S.* Senftenberg isolates from immunized and control group chickens. Number of CFU of *S.* Senftenberg strains, JOL1557, JOL1815, JOL1816 and JOL1817 per gram of faecal samples from chickens orally challenged with 5 × 10^8^ CFUs. Bacterial recovery was performed from day 1 to 28 post-challenge by direct plating and enrichment culture. Total 6 faecal samples were collected from six birds from each sub-group within major group A, B and C and processed for bacterial recovery. The data represented is mean CFU/gm ± SEM. * symbol indicates a significant amount of bacterial recovery from control group chickens compared to single and double immunized chickens

The fecal colonization of the JOL1557, JOL1815, JOL1816, and JOL1817 from the challenged chickens was evaluated to determine the protective efficacy offered by JOL1587 immunization. The fecal samples obtained from the immunized and control group chickens showed direct and post-enrichment recovery of all the challenge strains on BGA plates. The colonization and persistence of different S. Senftenberg was studied by challenging immunized as well as control group chickens. We divided each major group A, B and C post immunogenicity study into 4 sub-groups with six birds each. Each sub-group was challenged with different wild type *S.* Senftenberg (Table 2). Post-challenge bacterial recovery from fecal samples was performed to assess the protective efficacy. The bacterial recovery data was expressed as mean CFU/gm of each *S.* Senftenberg strain recovered from each sub-group (Fig. 5). Individual variation in the CFU recovery data was observed, hence mean value was calculated for each sub-group. The control group chickens showed significantly higher recovery of *S.* Senftenberg JOL1557, JOL1815, JOL1816 and JOL1817 strain until day 9, 28, 28 and 21 than prime-boost group, respectively (GI. 5, *p* < 0.05). Within immunized group, only the prime-boost immunized group showed significantly lower and below detection limit recovery for JOL1557, JOL1815, JOL1816 and JOL1817 from day 14 post-challenge (Fig. 5; *p* < 0.05).

Some chickens showed bacterial recovery only after enrichment culture; therefore, we interpreted the enrichments and direct recovery data as positive after for infected and shedding chickens and negative for non-shedding chickens. The percent population of positive (infected and shedding) and negative (non-shedding) chickens for *S*. Senftenberg infection are shown in Table 1. Total 80 to 100% chickens from the non-immunized control group showed fecal recovery of the JOL1557, JOL1815, JOL1816, and JOL1817 challenge strains consistently up to 4 weeks post-challenge, whereas the immunized chickens showed a significant decrease in the percentage population of the chickens positive for fecal shedding of the challenge strains (Table 2). Among the JOL1587 immunized chickens, the double immunized group showed significantly higher protection against *S.* Senftenberg than the single immunized and control group chickens (Table 2). Chickens from the double immunized group showed reduction in the challenge strain shedding below the detection level in the feces from day 9 post-challenge onward (Table 2). Although the single immunized group showed a significant reduction in the number of chickens positive for fecal shedding of the challenge strains, this group failed to show complete reduction in the shedding of wild–type *S.* Senftenberg below the detection limit from all the chickens (Table 2).

## Discussion

Contrary to other *Salmonella* serovars, the pattern of *S.* Senftenberg infection in chickens is characterized by colonization of the intestinal epithelium with very little induction of the systemic immune response [18]. Information regarding the quality and magnitude of the immune response needed for optimal protection against *S.* Senftenberg infection is sparse, and many factors still need to be investigated. Previously we developed a *S.* Senftenberg mutant strainJOL1587 *(Δlon and ΔcpxR)* for immunization of the chickens against wild-type S. Senftenberg infection [11]. In this study, we evaluated the magnitude and quality of immune response generated by JOL1587 immunization in chickens. Further, the generated immune response was evaluated for its ability to protect chickens against four different wild-type isolates of *S.* Senftenberg.

Generation of *Salmonella-*specific IgG and sIgA antibodies is essential for the development of acquired immune protection against NTS [19]. Because *S.* Senftenberg colonizes and persists in the cecum of chickens, the induction of a sIgA antibody response might be beneficial for the mucosal clearance of *S.* Senftenberg. *Salmonella*-specific sIgAs in the intestinal mucosa constitute a first line of defense by preventing bacterial penetration of the intestinal barrier through the mechanism of immune exclusion [20, 21]. In this study, we evaluated the potential of single and double immunization to induce a plasma IgG and intestinal sIgA response in chickens. From the ELISA results, it was evident that double immunization induced significantly higher IgG and sIgA levels than single immunization. It is possible that the booster immunization activates memory B cells, producing an increase in antibody titer by their rapid proliferation and differentiation into plasma cells [22]. The observed decline in the magnitude of IgG and sIgA levels in the single immunized group contrary to the double-immunized group may have been due to poor systemic invasion and lower levels of colonization in the spleen. This has been observed previously after experimental parenteral inoculation with *S.* Senftenberg in chickens [7]. The ELISA results highlight the necessity of a booster immunization in this case with JOL1857 to induce better humoral and mucosal immune responses against *S.* Senftenberg infection in chickens.

Along with humoral immunity, the generation of a *Salmonella*-specific cellular immune response (CMI) is a prerequisite for the establishment of immune protection against *Salmonella* spp. in chickens [23]. The production of CD4 and CD8 T cells as part of an adaptive cellular immune response is crucial for the clearance of *Salmonella* infection in mice [24]. Activated *Salmonella*-specific CD4 and CD8 T cells migrate to infected non-lymphoid tissues and exert their effector functions to enable *Salmonella* clearance [25]. In the present study, the single and double immunized chickens showed a significant increase in their LPA and CD8 T cell responses relative to control group chickens, but failed to induce a CD4 T cell response after primary immunization with JOL1857. Following oral inoculation of attenuated *Salmonella*, the antigen-specific CD4 T cell response is initially generated in the gut-associated lymphoid tissue (GALT), a primary site for processing intestinal immunogens in mice [26]. Because *S.* Senftenberg serovars have been shown to colonize intestinal epithelial cells following oral inoculation in chickens [18], the *Salmonella*-specific CD4 and CD8 T cell responses observed in this study after primary immunization with JOL1857 may be induced in the GALT of the chickens. The fact that intestinal lymphoid tissue possesses very few naïve CD3 + CD4+ T cells could explain the low T helper cell response after primary immunization with JOL1587 [27]. Only after the booster immunization was a significant increase in the CD4 T-cell level observed in the double immunized chickens. This significant increase in the CD4 T cell levels observed following booster immunization correlates with the increased antibody titers in the double immunized chickens. *Salmonella*-specific antibodies have been shown to upregulate the cellular immune response by amplifying the processing and presentation of antigens from *Salmonella* to T helper cells in mice [28, 29]. Overall, the prime-boost chickens immunized with JOL1857 displayed a significant increase in the *S*. Senftenberg-specific CMI response compared to the single immunized chickens.

To further elucidate the role of the CMI response in *S.* Senftenberg clearance from chickens, we investigated differences in the expression patterns of the cytokines IL-2, IFN-γ and IL-6 for immunized and control groups. The chickens immunized with JOL1587 showed significant upregulation of IFN-γ, IL-2 and IL-6 after receiving the primary and booster immunizations. As studied in mice infection model, the mechanisms employed by IFN-γ, IL-2 and IL-6 for establishing protection against *Salmonella* infection are diverse in nature [30–32]. The cytokines IL-2 and IFN-γ are known to mediate immune protection against *Salmonella* by up-regulating the generation of *Salmonella*-specific cell-mediated and mucosal immunity, respectively [33, 34]. The production of IFN-γ in the intestinal mucosa is essential for the generation of mucosal immunity against *Salmonella* infection [34]. In the absence of IFN-γ in the mucosa, infection with *Salmonella* Typhimurium has been shown to produce increased invasion and disseminated infection with septicemia in mice [34]. The multifunctional cytokine IL-6 has also been shown to confer a protective effect at the mucosal level against invasive *Salmonella* infection [32, 35]. Upregulation in IL-6 levels can trigger the rapid development of a cytotoxic T cell response against *Salmonella* infection in mice [36, 37]. Our results showed a correlation between an increase in IL-6 and the production of *S.* Senftenberg-specific CD8 T cells after booster immunization in the double immunized group. Collectively, the results from the immunogenicity study for JOL1587 in chickens suggest that the prime-boost immunization protocol can induce significant humoral, mucosal, and cellular immune responses against the *S.* Senftenberg serovar in chickens.

Current evidence suggests that optimal protection against *Salmonella* infection is conferred by vaccines that produce a pattern of immune response homologous to that induced by natural infection [38]. Hence, the ability of the immune response generated in response to JOL1587 immunization to establish immune protection was evaluated by challenging the chickens orally with four different wild-type strains of *S.* Senftenberg. Systemic infection with wild-type *S.* Senftenberg in chickens was reported to be self-limiting in nature [9]. Heterogeneity regarding caecal persistence and fecal shedding for various *S.* Senftenberg strains in chickens represents a potential challenge for this serovar’s suitability as a vaccine candidate [7]. The wild-type S. Senftenberg strains used in this study were tested positive for presence of SPI1, 2, 3, 4, and 5 specific gene with persistence in cecum of chicken. Evaluation of fecal shedding for the different challenge strains of wild-type *S.* Senftenberg can provide a useful means of estimating the protective efficacy conferred by JOL1587. The fecal shedding data from the protective efficacy experiments showed that, compared to the single immunization protocol, the prime-boost immunization with JOL1587 was sufficient for reduction of the wild-type *S.* Senftenberg recovery below the detection level in the feces of the chickens. Overall, the immunity generated in chickens by prime-boost immunization with JOL1587 was sufficient to protect chickens against infection with various wild-type *S.* Senftenberg strains.

## Conclusion

Our results indicate that chickens double immunized with JOL1587 can generate significant *S.* Senftenberg-specific mucosal, humoral and cell-mediated immune responses in chickens. Fecal shedding of the various *S.* Senftenberg wild-type isolates was significantly reduced below detection limit only in the double immunized chickens. In conclusion, the prime-boost immunization strategy of chickens with JOL1587 can provide a valuable method to prevent *S.* Senftenberg infection and fecal shedding in chickens.

## Abbreviations

APC: Allophycocyanin
CDC: Centers for disease control and prevention
CFU: Colony forming units
ELISA: Enzyme linked immunosorbent assay
FITC: Fluorescein-isothiocyanate
NTS: Non-typhoidal serotypes
OMP: Outer membrane protein
PBMC: Peripheral blood mononuclear cell
PE: Phycoerythrin
*S*. Senftenberg: *Salmonella* Senftenberg
sbcp: Sonicated bacterial cell protein

## References

[1] L’Ecuyer PB, Diego J, Murphy D, Trovillion E, Jones M, Sahm DF (1996). Nosocomial outbreak of gastroenteritis due to Salmonella senftenberg. Clin Infect Dis.

[2] Mohle-Boetani JC, Farrar JA, Werner SB, Minassian D, Bryant R, Abbott S (2001). Escherichia coli O157 and Salmonella infections associated with sprouts in California, 1996–1998. Ann Intern Med.

[3] Pezzoli L, Elson R, Little CL, Yip H, Fisher I, Yishai R (2008). Packed with Salmonella--investigation of an international outbreak of Salmonella Senftenberg infection linked to contamination of prepacked basil in 2007. Foodborne Pathog Dis.

[4] Rushdy AA, Stuart JM, Ward LR, Bruce J, Threlfall EJ, Punia P (1998). National outbreak of Salmonella senftenberg associated with infant food. Epidemiol Infect.

[5] Hamada S, Hashimato H, Tasaka T, Tsuchiya Y (1959). Studieson chick salmonellosis: II. Salmonella Senftenberginfections in chicks. Jpn. J. Vet. Res. Hokkaido University.

[6] Hu Q, Coburn B, Deng W, Li Y, Shi X, Lan Q (2008). Salmonella enterica serovar Senftenberg human clinical isolates lacking SPI-1. J Clin Microbiol.

[7] Boumart Z, Roche SM, Lalande F, Virlogeux-Payant I, Hennequet-Antier C, Menanteau P (2012). Heterogeneity of Persistence of Salmonella enterica Serotype Senftenberg Strains Could Explain the Emergence of this Serotype in Poultry Flocks. Roop RM, editor. PLoS ONE.

[8] Pedersen TB, Olsen JE, Bisgaard M (2008). Persistence of Salmonella Senftenberg in poultry production environments and investigation of its resistance to desiccation. Avian Pathol Taylor & Francis Group.

[9] Kallapura G, Kogut MH, Morgan MJ, Pumford NR, Bielke LR, Wolfenden AD (2014). Fate of Salmonella Senftenberg in broiler chickens evaluated by challenge experiments. Avian Pathol.

[10] Griffin AJ, McSorley SJ (2011). Development of protective immunity to Salmonella, a mucosal pathogen with a systemic agenda. Mucosal Immunol Society for Mucosal Immunology.

[11] Kamble NM, Lee JH (2016). Characterization and evaluation of *Salmonella enterica s* erotype Senftenberg mutant created by deletion of virulence-related genes for use as a live attenuated vaccine. Clin Vaccine Immunol.

[12] Kim SW, Moon KH, Baik HS, Kang HY, Kim SK, Bahk JD (2009). Changes of physiological and biochemical properties of Salmonella enterica serovar Typhimurium by deletion of cpxR and lon genes using allelic exchange method. J Microbiol Methods.

[13] Porter RE, Holt PS (1992). Use of a pilocarpine-based lavage procedure to study secretory immunoglobulin concentration in the alimentary tract of White Leghorn chickens. Avian Dis.

[14] Rana N, Kulshreshtha RC (2006). Cell-mediated and humoral immune responses to a virulent plasmid-cured mutant strain of Salmonella enterica serotype gallinarum in broiler chickens. Vet Microbiol.

[15] Chaudhari AA, Jawale CV, Kim SW, Lee JH (2012). Construction of a Salmonella Gallinarum ghost as a novel inactivated vaccine candidate and its protective efficacy against fowl typhoid in chickens. Vet Res BioMed Central.

[16] Livak KJ, Schmittgen TD (2001). Analysis of relative gene expression data using real-time quantitative PCR and the 2(−Delta Delta C(T)) Method. Methods.

[17] Nandre RM, Matsuda K, Chaudhari AA, Kim B, Lee JH (2012). A genetically engineered derivative of Salmonella Enteritidis as a novel live vaccine candidate for salmonellosis in chickens. Res Vet Sci.

[18] Boumart Z, Roche SM, Lalande F, Virlogeux-Payant I, Hennequet-Antier C, Menanteau P (2012). Heterogeneity of persistence of Salmonella enterica serotype Senftenberg strains could explain the emergence of this serotype in poultry flocks. PLoS ONE.

[19] MacLennan CA, Gondwe EN, Msefula CL, Kingsley RA, Thomson NR, White SA (2008). The neglected role of antibody in protection against bacteremia caused by nontyphoidal strains of Salmonella in African children. J Clin Invest.

[20] Holmgren J, Czerkinsky C (2005). Mucosal immunity and vaccines.

[21] Wijburg OLC, Uren TK, Simpfendorfer K, Johansen F-E, Brandtzaeg P, Strugnell RA (2006). Innate secretory antibodies protect against natural Salmonella typhimurium infection. J Exp Med.

[22] Galen JE, Chinchilla M, Pasetti MF, Wang JY, Zhao L, Arciniega-Martinez I (2009). Mucosal immunization with attenuated Salmonella enterica serovar Typhi expressing protective antigen of anthrax toxin (PA83) primes monkeys for accelerated serum antibody responses to parenteral PA83 vaccine. J Infect Dis.

[23] Penha Filho RAC, Moura BS, De Almeida AM, Montassier HJ, Barrow PA, Berchieri JA (2012). Humoral and cellular immune response generated by different vaccine programs before and after Salmonella Enteritidis challenge in chickens. Vaccine.

[24] Hess J, Ladel C, Miko D, Kaufmann SH (1996). Salmonella typhimurium aroA- infection in gene-targeted immunodeficient mice: major role of CD4+ TCR-alpha beta cells and IFN-gamma in bacterial clearance independent of intracellular location. J Immunol.

[25] Kirby AC, Sundquist M, Wick MJ (2004). In vivo compartmentalization of functionally distinct, rapidly responsive antigen-specific T-cell populations in DNA-immunized or Salmonella enterica serovar Typhimurium-infected mice. Infect Immun.

[26] McSorley SJ, Asch S, Costalonga M, Reinhardt RL, Jenkins MK (2002). Tracking salmonella-specific CD4 T cells in vivo reveals a local mucosal response to a disseminated infection. Immunity.

[27] Moon JJ, Chu HH, Pepper M, McSorley SJ, Jameson SC, Kedl RM (2007). Naive CD4(+) T cell frequency varies for different epitopes and predicts repertoire diversity and response magnitude. Immunity.

[28] Barr TA, Brown S, Mastroeni P, Gray D (2010). TLR and B cell receptor signals to B cells differentially program primary and memory Th1 responses to Salmonella enterica. J Immunol.

[29] Ugrinovic S, Ménager N, Goh N, Mastroeni P (2003). Characterization and development of T-Cell immune responses in B-cell-deficient (Igh-6(−/−)) mice with Salmonella enterica serovar Typhimurium infection. Infect Immun.

[30] Matsui K (1999). Role of interleukin-2 receptor expression on macrophages from Salmonella-infected mice. FEMS Immunol Med Microbiol.

[31] Muotiala A, Mäkelä PH (1990). The role of IFN-gamma in murine Salmonella typhimurium infection. Microb Pathog.

[32] Weinstein D, O’Neill B, Metcalf E (1997). Salmonella typhi stimulation of human intestinal epithelial cells induces secretion of epithelial cell-derived interleukin-6. Infect Immun.

[33] Paetkau V, Shaw J, Caplan B, Mills GB, Lee KC (1980). Interleukin 2 in cell-mediated immune responses. J Supramol Struct.

[34] Bao S, Beagley KW, France MP, Shen J, Husband AJ (2000). Interferon-gamma plays a critical role in intestinal immunity against Salmonella typhimurium infection. Immunology.

[35] Huang F-C (2009). Upregulation of Salmonella-induced IL-6 production in Caco-2 cells by PJ-34, PARP-1 inhibitor: involvement of PI3K, p38 MAPK, ERK, JNK, and NF-kappaB. Mediators Inflamm.

[36] Salazar-Gonzalez R-M, Srinivasan A, Griffin A, Muralimohan G, Ertelt JM, Ravindran R (2007). Salmonella flagellin induces bystander activation of splenic dendritic cells and hinders bacterial replication in vivo. J Immunol.

[37] Salerno-Goncalves R, Sztein MB (2009). Priming of Salmonella enterica serovar typhi-specific CD8(+) T cells by suicide dendritic cell cross-presentation in humans. PLoS ONE.

[38] Viret JF, Favre D, Wegmüller B, Herzog C, Que JU, Cryz SJ (1999). Mucosal and systemic immune responses in humans after primary and booster immunizations with orally administered invasive and noninvasive live attenuated bacteria. Infect Immun.

